# Quality of life in pulmonary arterial hypertension

**DOI:** 10.1007/s12471-015-0671-z

**Published:** 2015-03-04

**Authors:** M.C. Post, J.J. Mager

**Affiliations:** Department of Cardiology and Pulmonology, Center for Pulmonary Vascular Disease, St. Antonius Hospital, 3435 Nieuwegein/Utrecht, The Netherlands

In the current issue of The Netherlands Heart Journal, Blok et al. describes the quality of life (QoL) in patients with pulmonary arterial hypertension (PAH) due to congenital heart disease (CHD) [1].

In this observational study, 39 out of 85 patients (46 %) with PAH-CHD who received PAH-targeted therapy filled in the Short-Form Health Survey (SF-36) QoL questionnaire correctly and could be included. Out of these 39 patients, 50 % had Down syndrome. The diagnosis of PAH was based on echocardiography and defined as a tricuspid regurgitation velocity of at least 2.9 m/s (estimated systolic pulmonary artery pressure (PAP) of 36 mmHg) [1].

PAH is a subgroup of pulmonary hypertension, including different forms with the same histopathological changes primarily affecting the pulmonary arteries, leading to endothelial dysfunction, proliferative changes and vascular remodelling [2]. The prevalence of PAH is between 15 and 50 cases per million [3]. The diagnosis should be confirmed by right heart catheterisation, and is characterised by a mean PAP of at least 25 mmHg at rest and a pre-capillary haemodynamic profile including a pulmonary artery wedge pressure of less than 15 mmHg and a pulmonary vascular resistance of more than 3 Woods Units [2]. Echocardiography is used to estimate the systolic PAP based on the tricuspid regurgitation peak velocity and the presence of additional echocardiographic variables suggestive of pulmonary hypertension. The presence of pulmonary hypertension is likely if the velocity is more than 3.4 m/s (estimated systolic PAP > 50 mmHg) with or without additional variables [2]. Both underestimation and overestimation of the estimated systolic PAP frequently occur.

PAH is a debilitating and progressive disease with a tremendous psychosocial and economic impact for patients. A high prevalence of anxiety and depression (up to 40 %) is reported in patients with PAH leading to impairment of QoL [4]. An increase in clinical severity of the disease is associated with greater limitations and decrease in QoL. Exercise capacity, symptoms of right heart failure, haemodynamic characteristics, functional class and also mental disorders are associated with QoL. The majority of the studies on QoL in PAH patients used different standard generic questionnaires or tools specific for heart or lung diseases, not reflecting the clinical status of patients with PAH—for example the Minnesota Living with Heart Failure or the 36-item Medical Outcome Study Short-Form Health Survey Questionnaire (SF-36). Recently, a more disease-specific instrument was designed, the Cambridge PH Outcome Review (CAMPHOR) scale [5]. This scale exhibits superior psychometric properties, compared with the SF-36, in the assessment of pulmonary hypertension reported outcome [6].

In the study by Blok et al., almost all patients were on monotherapy receiving bosentan during follow-up [1]. In the 13 patients in whom the QoL declined during follow-up, the baseline functional class and exercise capacity were significantly worse, indicating more severe disease. No haemodynamic data (pulmonary vascular resistance or cardiac index) at baseline or changes in functional class during follow-up were reported. The 5-year survival rate was 82 % and age, baseline functional class or exercise capacity and decline in QoL appeared to be predictors for mortality in this study. It is important to know that a decline in QoL is associated with worse outcome, i.e. higher mortality in PAH-CHD. However, it remains unclear whether the use of serial QoL questionnaires is superior or additive to the current follow-up strategy of evaluation of functional class, biomarkers and exercise capacity.

Besides general measures and supportive therapy, targeted PAH therapy is indicated in symptomatic patients with pulmonary vascular disease and a decreased functional capacity (Modified New York Heart Association functional class of at least II) [2, 7]. The medical treatment of PAH is based on goal-oriented therapy [2]. The clinical response should be reassessed 3–6 months after the initial treatment and is based on different parameters or goals, such as: functional class, exercise capacity, biomarkers, echocardiography and haemodynamic parameters. If there is no improvement, sequential combination therapy should be started including another class of drugs [2]. Patients who achieve the treatment goals, irrespective of the type of medication used, seems to have a better prognosis and QoL. Predictors for outcome of PAH are sex, functional class, aetiology of PAH, exercise capacity, haemodynamics related to right ventricle performance and QoL [8].

CHD patients with an unrestricted pressure and volume overload due the presence of an intra- or extra-cardiac shunt are at risk for the development of PAH. However, PAH can develop at any stage of CHD, in the presence of a left-to-right shunt but also up to years after a corrective procedure [9]. The extreme end of the PAH-CHD spectrum is the Eisenmenger syndrome, characterised by a reversed (right-to-left) or bidirectional shunt with severe cyanosis and multiorgan involvement. It is a rare disease and the number of patients will decrease in the next decades due to the improvement of CHD treatment in recent decades. Recent recommendations have stated that the defect should only be repaired if no pulmonary vascular disease is present, i.e. an indexed pulmonary vascular resistance of less than 4 Woods Units × m^2^ [10, 11]. The estimated prevalence of PAH in adults with CHD is 10 %, and it has an adverse impact on outcome and QoL [12]. Besides PAH-targeted therapy, individualised rehabilitation programmes are indicated for PAH-CHD patients to improve QoL by enabling participation in social activities and to maintain an active lifestyle [2, 11]. Training leads to higher levels of physical activity, improved cardiorespiratory function and patient-reported QoL [13].

In patients with Down syndrome the prevalence of CHD, mainly atrioventricular septal defects, is about 50 %. These patients might be more prone to develop PAH compared with non-Down syndrome individuals with the same CHD. PAH-specific therapy seems to be safe and leads to increased exercise tolerance. However, the validity of measuring exercise capacity and QoL in this specific subgroup remains a concern [10].

Current guidelines on adult CHD and pulmonary hypertension recommend that patients with PAH-CHD should be followed by tertiary centres combining expertise in CHD and PAH in a multidisciplinary approach [2, 11].

## Conclusion

PAH related to CHD is a severe disease with a significant impact on the QoL, as demonstrated by Blok et al. [1]. It appears that the serial measurements of QoL using validated questionnaires might be of additive value and a decline in QoL seems to be associated with an increased mortality in PAH-CHD.
